# Positive expectations predict improved mental-health outcomes linked to psychedelic microdosing

**DOI:** 10.1038/s41598-021-81446-7

**Published:** 2021-01-21

**Authors:** L. S. Kaertner, M. B. Steinborn, H. Kettner, M. J. Spriggs, L. Roseman, T. Buchborn, M. Balaet, C. Timmermann, D. Erritzoe, R. L. Carhart-Harris

**Affiliations:** 1 Centre for Psychedelic Research, Division of Psychiatry, Imperial College London, London, UK; 2 Departmant of Psychology, Julius-Maximilans-University Würzburg, Würzburg, Germany; 3 Computational, Cognitive and Clinical Neuroimaging Laboratory, Imperial College London, London, UK

**Keywords:** Human behaviour, Quality of life, Placebo effect, Psychology

## Abstract

Psychedelic microdosing describes the ingestion of near-threshold perceptible doses of classic psychedelic substances. Anecdotal reports and observational studies suggest that microdosing may promote positive mood and well-being, but recent placebo-controlled studies failed to find compelling evidence for this. The present study collected web-based mental health and related data using a prospective (before, during and after) design. Individuals planning a weekly microdosing regimen completed surveys at strategic timepoints, spanning a core four-week test period. Eighty-one participants completed the primary study endpoint. Results revealed increased self-reported psychological well-being, emotional stability and reductions in state anxiety and depressive symptoms at the four-week primary endpoint, plus increases in psychological resilience, social connectedness, agreeableness, nature relatedness and aspects of psychological flexibility. However, positive expectancy scores at baseline predicted subsequent improvements in well-being, suggestive of a significant placebo response. This study highlights a role for positive expectancy in predicting positive outcomes following psychedelic microdosing and cautions against zealous inferences on its putative therapeutic value.

## Introduction

Classic tryptamine psychedelics are structurally related to the endogenous neurotransmitter serotonin (5-HT; 5-hydroxytryptamine) and induce their distinct psychological and physiological effects mainly through agonism of the 5-HT2A receptor^1,2^. In recent years, the phenomenon of psychedelic ‘microdosing’ has seen a significant rise in popularity and prevalence in western societies^3–5^. Generally, microdosing describes the frequent (e.g., near daily) intake of sub-threshold or threshold perceptible amounts of psychedelic substances. One of the most commonly described microdosing regimens involves dosing with a psychedelic every third or fourth day (e.g., 2 times per week) over a period of a few weeks^6^. Recommended and commonly used dose ranges lie between 5 and 20 μg of lysergic acid diethylamide (LSD), or 0.1–0.5 g of dried psilocybin containing mushrooms (e.g., psilocybe cubensis)^6^. However, there is no scientifically established definition on what microdosing entails or what constitutes a typical, or indeed effective, microdose^7,8^.

The growing popularity and media visibility of microdosing was brought into prominence by James Fadiman (2011), followed by expanding internet community interest^9,10^. The dominant motivation for microdosing appears to be a desire for positive changes in mood, or general well-being and cognitive enhancement, without acute subjective intoxication and associated behavioural disruption^11–15^. Microdosing is a quite different phenomenon to the single ‘full-dose’ administrations currently in clinical development^16,17^, where the typical protocol is to administer just one or two large doses (e.g., 25 mg) of psilocybin in guided clinical settings with careful context manipulation (e.g., participant screening and psychological support before, during and after the sessions), with the intention of engendering transformative psychological experiences and associated lasting improvements in mental health outcomes^18,19^. Important findings in this regard include evidence that just a few moderate to large doses of a psychedelic can produce enduring positive changes in outlook and behaviour in healthy volunteers^18,20–23^, as well as reduced psychiatric symptom severity in clinical populations^17,24–29^. Despite these positive findings, however, it is well known that high doses of psychedelics can induce psychologically challenging reactions including panic and/or psychotic states, particularly when the surrounding contextual conditions are adverse^19,20,30–33^. The increasing interest in microdosing in popular culture may have emerged as way of mitigating some of the perceived psychological challenges and risks associated with higher doses of psychedelics.

Anecdotal reports claim that sub-threshold/threshold perceptible psychedelic microdoses can have beneficial sub-acute effects on psychological functioning and well-being despite having negligible acute (subjective) psychoactivity^6^. Until now, the effects of microdosing have mainly been investigated using observational surveys^11,13,14,34^ and open-label studies^35^. While mindful of the methodological limitations of uncontrolled studies such as these, the findings are largely supportive of positive anecdotes regarding microdosing, i.e., results have largely found positive effects on mental-health outcomes^11,13,14,34^ and cognition^35^. However, a general positive test strategy, and various components of confirmation bias—including visible demand characteristics, positive expectancy and self-selection—may combine to increase the likelihood and magnitude of positive outcomes. Tellingly, recent attempts at placebo-controlled studies on microdosing in healthy volunteers with double-blind drug administration have failed to find compelling evidence for beneficial effects of microdoses on cognition or mood, as compared with placebo^36–38^, and participants in these studies were able to detect subjective drug effects, thus jeopardising the effectiveness of the placebo control^36–38^. This is a common issue in drug studies, particularly when the relevant drugs have discernible subjective effects. Ineffective blinding may corrupt the rigour of placebo-controlled studies, and increase the risk of experimental biases^39^. Positive expectancy may be an important contributor to positive outcomes following psychedelic drug use and thus, an important source of bias.

Investigating placebo or ‘enhanced placebo’ effects (where drug effects positively interact with positive psychological expectations) in psychedelic microdosing studies is important^40–43^. Typically, placebos are interventions with no direct activity but serve to control for positive (or negative, i.e. ‘nocebo’^44^) expectations linked to particular interventions^45^. Placebo effects may affect various outcomes (e.g., subjective experiences, symptoms, behaviour or physiological responses)^45^ and can be modulated by multiple contextual and psychobiological mechanisms^46^, whereas expectations about a treatment are among the most important factors contributing to the placebo response^46^. Generally, it can be assumed that expectations in the microdosing sub-culture are positively biased, as has been found in previous research^14^. Positive media coverage of the topic have likely contributed to this cultural bias^4,6,9,10,12^ and this may impinge on outcomes from microdosing and thus, the results of studies such as the present one.

Due to the pragmatic challenges of doing so via an online observational study, the present study did not include a placebo control condition. We did, however, employ a prospective, naturalistic design that included baseline sampling of expectations about possible outcomes from the impending microdosing. Well-being, state anxiety and depressive symptom scores were measured weekly on five occasions (pre-dosing at baseline to week 4 of the microdosing regimen) in order to track time-dependent changes. Neuroticism/emotional stability was measured pre-dosing at baseline and post-dosing at week 4 only. It was predicted that well-being and emotional stability would be increased, and that depression and anxiety scores would be decreased, at the key-endpoint (4 weeks) compared with baseline. Capitalising on the nature of the prospective design, we also predicted that baseline positive expectations about microdosing would be related to any subsequent improvements in well-being, depressive symptoms and anxiety scores. Finally, exploratory analyses were performed to assess pre-post changes in a range of secondary psychological outcomes of interest.

## Materials and method

### Design

The study was approved by the Imperial College Research Ethics Committee (ICREC reference 18IC4361) and was conducted in accordance with the framework of Good Clinical Practice (GCP). The study description and eligibility criteria were described on the study website and the informed consent procedure was also written in the initial home page for the study, and involved participants clicking to declare informed consent. The study weblink is here and relevant informed consent text can be found in an attached supplementary material document (Supplementary Methods).

Surveys were created within and hosted using the online survey platform Surveygizmo and an email notification system was managed by the website psychedelicsurvey.com. The sample consisted of a cohort of volunteers planning to start microdosing in the near future. Participants were not encouraged to microdose, but rather to register data pertaining to their pre-planned microdosing experience by participating in the study. Data were collected using web-based surveys at different time-points. Eligibility criteria included: being at least 18 years of age, having a good understanding of the English language and having the intention to microdose one of the following: psilocybin/magic mushrooms/truffles, LSD/1P-LSD, ayahuasca, DMT/5-MeO-DMT, salvia divinorum, mescaline, or iboga/ibogaine, for at least four weeks in the near future. Individuals who were already microdosing were excluded.

Participants were recruited online by disseminating advertisements with the link to the study webpage (www.microdosingsurvey.com) on several drug-related online-platforms^9,47^ in social media online communities (Facebook, Twitter) and via word of mouth. After declaring informed consent, individuals were able to sign up by providing their name, e-mail address, and the date on which they planned to start their microdosing protocol. Once registered, participants received e-mails via psychedelicsurvey.com containing links to the relevant questionnaires based on their indicated start date. Anonymity was ensured through the use of unique identifiers that could not be tracked back to personal information. Participants were not instructed in self-administration techniques or dosage frequencies and were thereby free to arrange their dosing routine ad libitum. This flexibility in administration procedure ensured congruency with their normal daily routine and naturalistic quality of the study.

In this prospective study, participants completed at least five surveys at different time points: one week before, and once weekly throughout the four-week time period over which they engaged in their individual microdosing protocol. Two additional measures at week 5 and week 6 were included in order to capture individuals who chose to microdose longer than four weeks. Two additional follow-up timepoints at six- and twelve-months post start date were included, but insufficient numbers had completed these at the time of analysis. Each survey consisted of a large, comprehensive battery of validated measures, plus a small number of self-constructed scales targeting specific concepts of interest.

### Measures

#### Survey 1: baseline

##### Timing and duration

The baseline survey was sent to participants one week before their indicated microdosing start date (if there was sufficient time) or immediately after they signed up. This survey took approximately 43 min to complete.

##### Demographic data

This first survey collected demographic information such as age, sex, nationality, native language, educational background, employment status, history of psychiatric illness, prior and current use of psychiatric medication, previous use of legal and illicit drugs and previous microdosing experience. We aimed to assess a potential sample bias by asking participants to specify their relationship to psychedelic substances according to a set of statements^20^ that were rated on a 5-point Likert scale*: *“I am an active advocate of psychedelic drug use”, “I am an active advocate of the therapeutic use of psychedelics”, “I have an advanced knowledge about psychedelics”, and “I am a highly experienced psychedelic drug user”.

##### Microdosing parameters and expectations

Participants were then asked to specify their plans for their upcoming microdosing protocol. These included the substance they planned to use (e.g., “psilocybin/magic mushrooms/truffles”, “LSD/1P-LSD”, “ayahuasca”, “DMT/5-MeO-DMT”, “salvia divinorum”, or the option to give a free answer), the number of dosing days per week, the planned dose and the duration of their microdosing protocol (4, 5 or 6 weeks).

Information on participants ‘expectations was gathered at baseline using four Visual Analogue Scales (VAS; 0–100) items derived from the credibility/expectancy questionnaire^48^: “How confident are you that the upcoming microdosing experience will have a long-lasting positive effect?” (0 = not at all confident, 50 = somewhat confident, 100 = very confident)*, *“At this point, how logical does the microdosing experience seem to you?” (0 = not at all logical, 50 = somewhat logical, 100 = very logical)*, *“At this point, how successfully do you think this experience will be in improving your overall well-being?” (0 = not at all useful, 50 = somewhat useful, 100 = very useful), and “By the end of the experience, how much improvement of your overall well-being do you think will occur?” (0–100 percent). An overall expectancy score was calculated by taking the mean of these four scales. Participants also stated if they had prior experience with microdosing (“yes”/“no”) and if they were currently microdosing (“yes”/“no”), in order to reliably exclude individuals that were already microdosing at the time of signing up for the study.

##### Outcome measures

All outcome measures used in the baseline survey are summarised below:

##### Primary outcome

The 14-item Warwick-Edinburgh Mental Well-being Scale (WEMWBS)^49^ served as the primary outcome measure for this study. The WEMWBS captures positive mental health and well-being. This questionnaire was used to track changes in well-being during the microdosing process. The WEMWBS covers hedonic and eudaimonic aspects of positive mental health, such as positive affect, psychological functioning and interpersonal relationships.

##### Clinically relevant variables

The 16-item Quick Inventory of Depressive Symptomatology (QIDS-SR_16_)^50^ is a self-report questionnaire and was included to capture depressive symptom severity and symptom change. State anxiety was assessed using the short form of the Spielberger State-Trait Anxiety Inventory (STAI-6)^51^.

##### Trait and trait-like variables

The Ten-Item Personality Inventory (TIPI)^52^ was included to measure the five personality domains known as the *Big Five*^53^, namely: openness to experience, extraversion, agreeableness, conscientiousness and emotional stability (i.e. inverted neuroticism). Trait absorption (i.e., being more susceptible to immersion in certain experiences) was measured using the modified version of the Tellegen Absorption Scale (MODTAS)^54^. The Short Suggestibility Scale (SSS) of the Multidimensional Iowa Suggestibility Scale (MISS)^55^ consists of 21 items capturing consumer and physiological suggestibility, persuadability, peer conformity and physiological reactivity. Additional scales were included to capture three distinguishable aspects of connectedness: (1) connection to self, (2) others and (3) nature. The 8-item Social Connectedness Scale (SCS)^56^ was included to assess connectedness to others in the social environment, and the 6-items Nature Relatedness Scale (NR-6)^57^ was used to capture relatedness to nature. The brief experiential avoidance questionnaire (BEAQ)^58^ was included as a measure of psychological flexibility, and indexes constructs such as unwillingness to remain in contact with distressing emotions, thoughts, memories and physical sensations (i.e. emotional acceptance). The brief resilience scale (BRS)^59^ was used to capture the ability to recover from stress. Lastly, the Peters et al. Delusions Inventory (PDI) was included to assess delusional ideation^60^.

#### Surveys 2,3 and 4: weekly measurements

##### Timing and duration

The weekly surveys were sent out at the end of weeks one, two and three post-start date, and took approximately 25 min to complete. Key measures of change included: the WEMWBS, QIDS-SR_16_, and STAI-6.

##### Subjective drug effects

Two measures that have been used in former studies to capture key-features of full-dose psychedelic experiences were included to assess the intensity of low dose psychedelic drug effects. The 11-Dimensional Altered States of Consciousness Rating Scale (11D-ASC)^61^ is a widely used measure of deviations from normal waking consciousness. For efficiency, eight of the eleven subscales of the 11D-ASC were included to capture the acute effects of psychedelic drugs. The Ego Dissolution Inventory (EDI)^62^ was included to assess altered ego-consciousness.

##### Microdosing parameters

Participants were asked to specify the microdosing particulars (drug type, number of dosing days during the last week, doses on each dosing day), which could be selected from default options. The doses were additionally specified manually (drug type and measuring unit). Two self-constructed items were included to assess the average dose (done by referencing doses to LSD equivalents) and average intensity of the drug effects of the preceding week, rated on a 6-point rating scale respectively. The reference to LSD was chosen to standardise responses due to the heterogenous substances that were used and capture potential changes in dose and drug effects during the course of microdosing: “What was the average amount of the drug you used on your dosing day/s?” with the following respone options: “Tiny microdose (LSD reference: 1–5 mcg or ~ 1/20 of a tab max*)”, “Small microdose (LSD reference: 6–10 mcg or ~ 1/10 of a tab max*)”, “Moderate microdose (LSD reference: 11–15 mcg or ~ 1/7 of a tab max*)”, “Moderate/’high’ microdose (LSD reference: 16–20 mcg or ~ 1/4 of a tab max*)”, “‘High’ microdose (LSD reference: 21–30 mcg or ~ 1/3 of a tab max*)”, “‘Very high’ microdose (LSD reference: 31 + mcg or more than 1/3 of a tab*)”. Subsequently, participants rated the average intensity of the subjective effects: “definitely no detectable effects”, “effects so slight, I could just be imagining them”, “possible mild effects”, “mild but quite noticeable effects”, “clearly noticeable effects”, “stronger than typical ‘microdose’ level effects”.

#### Survey 5: primary-endpoint

##### Timing and duration

The key-endpoint survey was sent out 4 weeks post start date and took approximately 50 min to complete.

##### Outcome measures

This survey re-evaluated all relevant outcome measures that were assessed at baseline. Microdosing characteristics and subjective drug effects (ASC subscales and EDI) were also included.

### Statistical analysis

Repeated measures analysis of variance (ANOVAs) with Greenhouse–Geisser corrections were conducted to evaluate time-dependent changes in the main outcome measures over all five time-points. The relevant questionnaire score was included as dependent variable and time as within-subject effect with 5 levels (baseline, week 1, 2, 3 And 4). a priori planned contrasts (Simple) were used to assess changes from baseline. To test the secondary hypothesis that expectancy scores would affect changes in the primary outcome measures (expectancy effect), one-tailed partial correlations using Pearson coefficient were conducted, testing the effects of baseline expectancy on endpoint change scores (endpoint—baseline scores), while controlling for baseline scores.

Finally, two-tailed dependent samples *t*-tests were applied to test for changes in the remaining outcome variables measured at baseline and endpoint. With the exception of changes in the personality facet emotional stability, there were no pre-defined hypotheses for changes in these measurs and results should be treated as explorative. Lastly, a supplementary analysis was conducted in order to further explore the effects of microdosing on depressive symptomatology (QIDS-SR_16_) in a clinically relevant subsample. The sample was split based on baseline QIDS-SR_16_ scores (depressed vs. non-depressed) and a mixed between-within ANOVA was employed to assess changes in depressive symptomology in the two groups across time. Results can be found in the supplementary material (Supplementary Methods). For all analyses, a significance threshold of *p* < 0.05 was specified. Analyses were carried out using IBM SPSS Statistics version 25 and R 3.6.3.

## Results

### Demographic & participant information

Descriptive statistics and Pearson correlations of relevant baseline measures can be found in Supplementary Table S1. Of 316 initial sign-ups, 63 participants were already microdosing and were thus excluded from the present analysis. At the time of analysis, *N*_*1*_ = 253 participants completed the baseline measure, with the subsequent weekly surveys completed by *N*_*2*_ = 162, *N*_*3*_ = 115, *N*_*4*_ = 102, and *N*_*5*_ = 81 participants. The mean age of the sample was 35.47(± 11.87) years and was predominantly male (60.5%). See Supplementary Table S2 for additional demographic information.

A total of 117 participants (46.2%) reported to have been diagnosed with one (or more) of the 15 specified psychiatric disorders, which were: major depressive disorder (MDD), bipolar disorder, schizophrenia, anxiety disorder, panic disorder, serious phobia (agoraphobia, social phobia), substance abuse disorder, alcohol dependence, hallucinogen persisting perception disorder, psychotic disorder, attention deficit hyperactivity disorder (ADHD), obsessive compulsive disorder (OCD), eating disorder, and post-traumatic stress disorder (PTSD). Additional 11 participants (4.3%) stated suffering from a self-diagnosed disorder or a disorder that was not specified in the responses (e.g., dysthymia, autism spectrum disorder). The most prevalent reported disorders across the sample population were major depressive disorder (*N* = 66, 26.1%) and anxiety disorder (*N* = 61, 24.1%) followed by ADHD (*N* = 27, 10.7%) and PTSD (*N* = 24, 9.5%).

Most participants were experienced psychedelic drug users; 215 participants (84.9%) reported that they had taken a classic psychedelic at least once in their life, and 38 (15%) were psychedelic drug naïve. Furthermore, 75 participants (29.6%) had experimented with microdosing prior to the study. Inspection of the four items assessing participants’ attitude towards psychedelic drugs revealed that the sample was positively biased towards psychedelic drug use in general and also towards therapeutic use of psychedelics, which was expected due to the nature of participant recruitment and self-selection bias (Supplementary Figure S1). A total expectancy score was calculated by taking the mean of the four expectancy items, ranging from 0 (absent positive expectancy) to 100 (extreme positive expectancy) (*M* = 65.10 ± 19.95).

### Microdosing parameters

Participants who completed all five time-points (from baseline to key-endpoint at 4 weeks, n = 68) had on average *M* = 9.2 (*SD* = 2.31, *range* = 4–18) dosing days during the study, and *M* = 2.31 (*SD* = 0.58, *range* = 1–5) dosing days per week.

At baseline, the majority of the 253 participants planned to use Magic Mushrooms/Psilocybin (*N* = 121, 47.82%), LSD, or LSD analogues such as 1P-LSD (*N* = 106, 41.9%). Seventeen participants (6.7%) planned to mix those two substances during their microdosing regime; 0.8% (*N* = 2) reported using DMT/5-MeO-DMT, 1.2% (*N* = 3) Mescaline and 1.6% (*N* = 4) Ibogaine. Microdosing parameters are shown in further detail in Supplementary Table S3.

### Changes in main outcome measures

The descriptive statistics of the main outcome measures at individual time-points are displayed in Table 1.

**Table 1:** Descriptive statistics for self-report data of main outcome variables over five time-points.

Variables	Timepoint	*M*	*SD*	Skewness
WEMWBS^a^	1	43.65	9.94	− .23
	2	48.19	9.35	− .48
	3	49.24	8.61	− .42
	4	49.51	8.68	− .67
	5	51.14	8.55	− .42
QIDS-SR^b^	1	9.25	5.73	.57
	2	5.09	4.01	1.9
	3	4.24	3.68	2.04
	4	3.82	3.45	1.77
	5	3.85	3.73	1.60
STAI-6^c^	1	48.70	14.61	.11
	2	40.06	13.28	.84
	3	38.29	12.09	.75
	4	37.78	12.64	.70
	5	37.98	10.59	.97

*N* = 68. This sample consists of individuals who completed all five time-points. *M* = mean; *SD* = standard deviation of mean and Skew = skewness are shown per TP = time-point (1 = baseline; 2–5 = week 1–4) for the Warwick-Edinburgh Mental Well-being Scale (WEMWBS), the Quick Inventory of depressive Symptomatology (QIDS) and the six-item short-form of the state scale of the Spielberger State-Trait Anxiety Inventory (STAI-6). Higher scores on the WEMWBS indicate better psychological well-being. Lower scores in the QIDS-SR and STAI-6 reflect lower depressive and anxiety symptoms, respectively.

^a^WEMWBS population norms in Health Survey for England 2011 = 51.61.

^b^QIDS-SR_16_ severity of depression: < 5 no depression, 6 to 10 mild depression, 11 to 15 severe depression, 16 to 20 very severe depression.

^c^Spielberger State-Trait Anxiety Inventory for Adults Manual; approximative normative scores for the age range of the present sample is ~ 36 (Spielberger, 1983).

#### Well-Being

**Figure 1:**
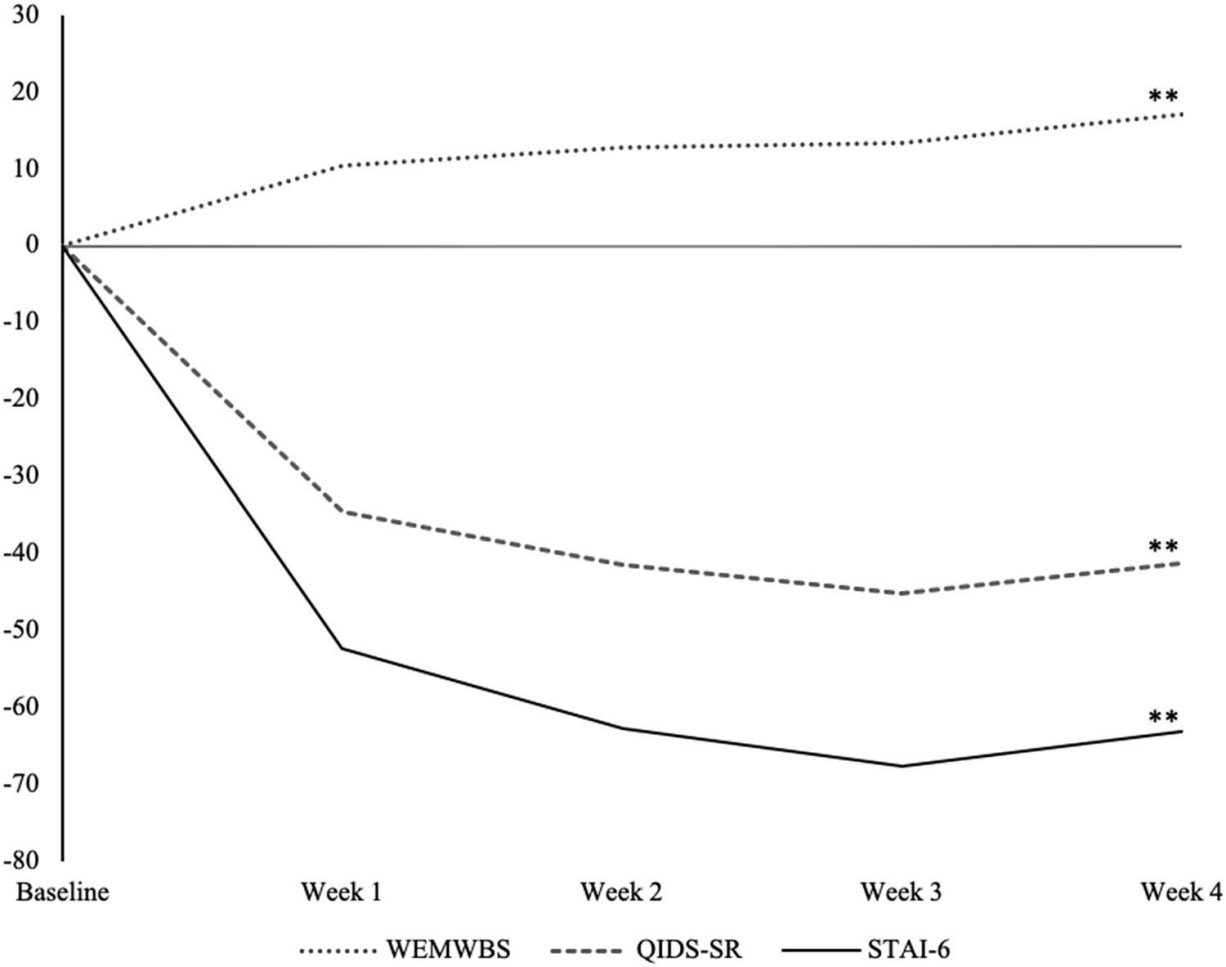
Percentual changes from baseline are shown for the Warwick-Edinburgh Mental Well-being Scale;(WEMWBS); Spielberger State-Trait Anxiety Inventory, short version (STAI-6); Quick Inventory of Depressive Symptomatology (QIDS-SR_16_). *N* = *68*. Note that percentage gains or losses indicate increases or reductions in absolute values. Results of repeated measures ANOVAs are shown with *p < .05, **p < .01.

There was a statistically significant increase in psychological well-being over time. This is indicated by the main effect of the factor ‘time’ on the well-being score [*F*(2.9, 191) = 14.441, *p* < 0.001, *η*_*p*_*2* = 0.18]. This increase was most pronounced at the second time-point (week 1) but quickly reached asymptote afterwards (Fig. 1). The planned contrast analysis (simple) showed significant differences between well-being scores at baseline and all successive timepoints: week 1 [*F*(1, 67) = 14.326, *p* < 0.001, *η*_*p*_*2* = 0.18], week 2 [*F*(1, 67) = 20.74, *p* < 0.001; *η*_*p*_*2* = 0.23], week 3 [*F*(1, 67) = 18.80, *p* < . 001; *η*_*p*_*2* = 0.22] and week 4 [*F*(1, 67) = 31.82; *p* < 0.001, *η*_*p*_*2* = 0.32]*.*

#### Severity of depressive symptoms

Results revealed a significant decrease in depressive symptoms over time; indicated by the main effect of the factor time on depressive symptoms [*F*(2.3, 120.7) = 23.702, *p* < 0.001, *η*_p_^2^ = 0.31]. This decrease was most pronounced at the second and third time-point (week 1 and week 2), but quickly reached asymptote afterwards (Fig. 1). The planned contrast analysis (simple) showed a significant difference between depressive symptoms scores at baseline and all subsequent time-points: week 1 [*F*(1, 67) = 29.854, *p* < 0.001, *η*_p_^2^ = 0.36], week 2 [*F*(1, 67) = 37.087, *p* < 0.001, *η*_p_^2^ = 0.41], week 3 [*F*(1, 67) = 34.801, *p* < 0.001, *η*_p_^2^ = 0.40] and week 4 [*F*(1, 67) = 33.179, *p* < 0.001, *η*_p_^2^ = 0.39].

#### Anxiety

Results revealed a significant decrease in state anxiety over time; indicated by the main effect of the factor time on the state anxiety scores [*F*(3.1, 205.8) = 20.755, *p* < 0.001, *η*_p_^2^ = 0.24]. This decrease was most pronounced at the second time-point (week 1), but also quickly reached asymptote afterwards (Fig. 1). The planned contrast analysis (simple) revealed a significant difference between baseline state anxiety scores and all subsequent time-points at week 1 [*F*(1, 67) = 31.425, *p* < 0.001, *η*_p_^2^ = 0.32], week 2 [*F*(1, 67) = 40.406, *p* < 0.001, *η*_p_^2^ = 0.38], week 3 [*F*(1, 67) = 29.989, *p* < 0.001, *η*_p_^2^ = 0.31] and week 4 [*F*(1, 67) = 50.638, *p* < 0.001, *η*_p_^2^ = 0.43].

### Expectancy effect on main outcome change scores

One-tailed partial correlations using Pearson coefficient were employed in order to investigate the effects of baseline expectations on endpoint change scores (endpoint—baseline) for the primary outcome variables (well-being, depressive symptoms and anxiety), whilst controlling for the corresponding baseline scores. In line with our main hypothesis, expectations for well-being improvement were significantly associated with change scores in well-being (*r* = 0.275, *p* = 0.007), depressive symptoms (*r* = − 0.263, *p* = 0.009) and anxiety (*r* = − 0.220, *p* = 0.025). These results indicate that baseline expectations were predictive of mental health change at the study endpoint.

### Exploratory analyses

**Figure 2:**
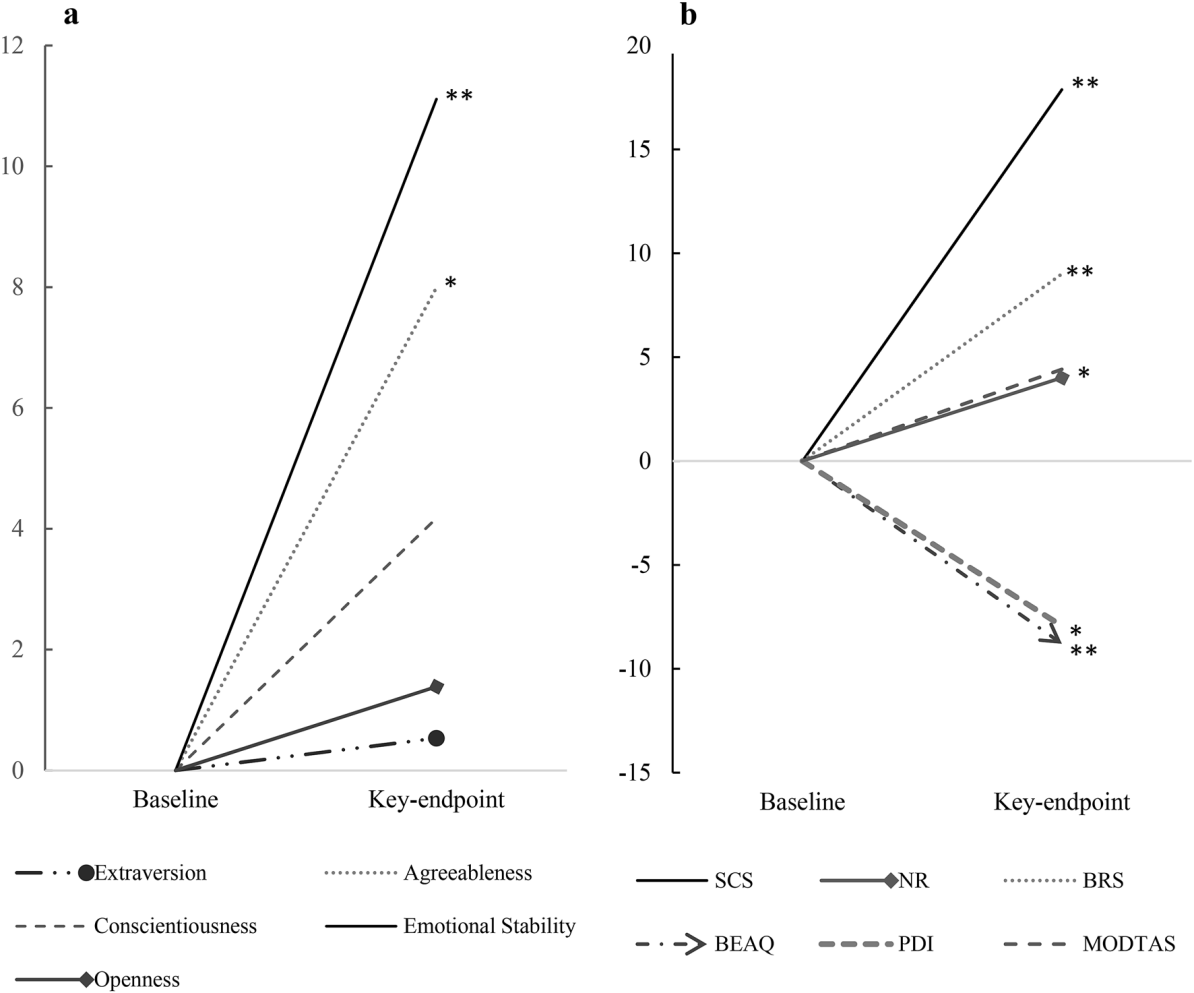
(**a**) Percentual changes from 1) baseline to 2) key-endpoint (4-weeks) are shown for the subscales of the Ten-Item Personality Inventory (TIPI): Extraversion, Agreeableness, Conscientiousness, Emotional Stability, Openness. (**b**) Percentual changes from 1) baseline to 2) key-endpoint (4-weeks) are shown for the subscales of the Social Connectedness Scale (SCS), Nature-Relatedness (NR-6), brief Resilience Scale (BRS), and Brief Experiential Avoidance Questionnaire (BEAQ). Note that percentage gains or losses indicate increases or reductions and increases in absolute values. N = 81, results of dependent samples t-tests are shown* *p* < .05, ** *p* < .01.

The following analyses were conducted with the total sample who completed all timepoints (*N* = 81). In line with our prior hypothesis, results revealed a significant increase in the personality facet emotional stability (*p* < 0.001, *d* = 0.40) (Fig. 2a). Other analyses can be considered exploratory and many of these outcome measures test inter-related constructs; as such, Bonferroni-correction was not applied. Results are summarised in Table 2 and visualised in Fig. 2b.

**Table 2:** Descriptive statistics and results of dependent samples t-tests for remaining outcome variables measured at baseline and key-endpoint.

Variables	Mean difference				
	*M*	*SD*	*t* (80)	*p*	Cohen’s *d*
BRS	.29	.62	4.25	< .001**	.47
BEAQ	− 3.84	10.03	− 3.44	.001**	.38
SCS	5.40	13.44	3.61	.001**	.40
NR-6	.16	.53	2.69	.009**	.29
TIPI-E	.02	1.10	.203	.840	.02
TIPI-A	.37	1.01	3.32	.001**	.37
TIPI-C	.20	.96	1.90	.061	.21
**TIPI-ES**	.46	1.15	3.64	< .001**	.40
TIPI-O	.09	.77	1.01	.318	.11
MODTAS	1.70	9.80	1.57	.122	.17
PDI	− .44	2.00	− 2.05	.044*	.23
SSS	− .84	6.58	− 1.15	.254	.12

*N* = 81. Results of two-tailed dependent samples t-tests are shown. *M* = mean difference (key-endpoint – baseline) ± *SD* = standard deviation of mean difference, Skew = Skewness; Negative change scores indicate a decrease, positive change scores an increase from baseline to key-endpoint at 4 weeks. Variables that were subject to specific hypotheses are marked in bold. Significant effects are denoted in **bold**, *p*-values and effect sizes (Cohen’s *d*) are shown. * *p* < .05, ** *p* < .01. Brief Resilience Scale (BRS); Brief Experiential Avoidance Questionnaire (BEAQ); Social Connectedness Scale (SCS); Nature Relatedness (NR-6); Ten-Item Personality Inventory (TIPI) with the subscales on I Extraversion, (A) Agreeableness, (C) Conscientiousness, (ES) Emotional Stability and (O) Openness; Modified Tellegen Absorption Scale (TAS), Peter’s Delusion Inventory (PDI) Short Suggestibility Scale (SSS). When Bonferroni adjusted alpha levels of .004 were applied for each test, the change in PDI was no longer significant. Descriptive statistics for both time-points can be found in the supplementary material document (Supplementary Table S4).

## Discussion

The current study provides the first prospective exploration of microdosing in naturalistic settings and is, to our knowledge, the first to highlight the role of positive expectations in predicting pertinent psychological outcomes linked to psychedelic drug use.

Consistent with previous reports and our own hypotheses, positive changes in well-being, depressive symptoms, state anxiety and emotional stability were observed following 4-weeks of microdosing. Further, positive changes in agreeableness, social connectedness, nature relatedness, resilience, delusional ideation, and psychological flexibility were observed in explorative secondary analyses.

On face value, like previous work^11,13,14,34^, the present findings could be viewed as another endorsement of the positive claims about microdosing; however, consistent with our main hypothesis, positive expectations measured at baseline were found to be significantly predictive of the main improvements in mental health observed at the key four week endpoint, namely: increased well-being, and decreased anxiety and depressive symptom scores.

The relationship between baseline positive expectation and subsequent outcomes is not unique to psychedelics^45,46,63,64^; however, it does highlight some important considerations for microdosing research, where placebo controlled microdosing studies have so far yielded mostly negative findings. The importance of including a placebo arm in future microdosing studies is an obvious implication of the present work but another, broader one, is that sampling the role of expectations holds value in psychedelic research more generally, as its contribution may be considerable. This issue is related to the often expressed influence of prior mind ‘set’ (including prior expectations), on the quality of a psychedelic experience and subsequent psychological outcomes^18,65^.

Most participants in the present study reported 2–3 [*M* = 2.30. *SD* = 0.58] dosing days per week and followed the so-called ‘Fadiman’—protocol that suggests the ingestion of a microdose once every three days, for several weeks^6^. This fidelity to the Fadiman protocol is interesting, considering that participants were not instructed on or ‘nudged’ toward a predefined routine, and instead, could arrange a flexible dosing routine in a way that suited their daily duties and activities. Importantly, participants were able to adapt their routine based on any effects (or side-effects) experienced—which may have promoted optimal effects and served convenience and retention (see Supplementary Table S5).

Merits of observational research, such as the present study, include its strong ecological validity and pragmatic flexibility; however, these strengths are counter-weighted by significant weaknesses linked to a lack of experimental control. In the present study, uncertainties and likely inaccuracies linked to drug dosage estimates, and inconsistent drug purity and potencies, are a particular limitation. In the absence of any previously used standard measurement of dose, we used ‘LSD equivalent dosages’ to calibrate dosage estimates. While this may have introduced additional inaccuracies for participants microdosing with non-LSD substances, approximately 40% of the sample did use LSD, and another ~ 50% used psilocybin containing mushrooms, for which online microdosing guidelines provide some conversion of dose. Empirical exploration of how best to customise parameters to optimise response and minimise side-effects is necessary to advance the field.

Another major limitation of the present study, also linked to its observational design, was the lack of a control or placebo group. This lack of control impairs our ability to make inferences about the causal effects of microdosing itself, above and beyond e.g. positive expectation or ‘placebo’ effects. So-called ‘regression to the mean’ effects (a statistical phenomenon due to random variance in the data) also requires some consideration, however, most participants did not present with extreme scores at baseline and well-being is known to be relatively stable in most populations^66,67^. The possibility that the present findings may in part be explained by spontaneous remission (an unexpected improvement of physical or psychological symptoms of a specific condition without the aid of a treatment), however, cannot be excluded. The high rates of attrition (with dropout rate of 68% at 4-weeks post start date) might have also created a systematic bias favouring those who experienced positive effects. Importantly, however, there was no evidence that early side-effects such as an increase in anxiety predicted drop-out at 4 weeks. Rather, lower baseline conscientiousness and young age were the only significant predictors of drop-out (Supplementary Methods). For future reference and comparisons, interested readers are directed to a list of potential side-effects of microdosing in the Supplementary Material (see the ‘Post-Treatment Changes Scale, PTCS, Supplementary Table 5).

As highlighted, perhaps the most novel and interesting finding of the present study was the discovery of a strong relationship between prior positive forecasts about the mental health benefits of microdosing and the benefits that were subsequently reported. The measure we employed to test expectancy was a modified version of the ‘credibility/expectancy’ questionnaire^48^. This measure was revised to focus on expectations about the long-term effects of microdosing. It would be interesting in the future to also assess relationships between prior expectations and acute effects – some of which are known to be strongly predictive of subsequent longer-term mental health outcomes. In the specific context of microdosing (but also more generally), we would hypothesise that expectations of impending drug effects may sensitise people to perceived changes in their conscious experience (whether ‘real’ or imagined). In some cases, highly-sensitive individuals may selectively attend to perceived (but imagined) changes in their conscious experience and mislabel them as ‘true’ drug effects^68^. Equally, ‘true’ drug effects may be felt that then go on to modulate expectations in a dynamic way. Prior experience with psychedelics may contribute to both phenomena, and since microdosing typically involves repeat administration, it is logical to surmise that expectations could change over the duration of a course of microdosing. Sampling such dynamic changes might be another interesting area for future research.

It seems entirely plausible that microdoses function as ‘active placebos’ amplifying expectations due to the (e.g. plasticity-promoting) nature of the drug effects themselves^36,37,69–71^. Indeed, such a possibility is supported by pharmacological evidence, including the evidence-backed assumption that psychedelic experiences are not only highly context-dependent but that also that they actively enhance context sensitivity^19,43,72^. In randomised placebo-controlled trials (RCTs), it is usually assumed that key confounding variables are consistent across conditions, and that any subsequent differences in between-group contrasts can be ascribed to the pharmacological action of the experimental drug of interest^40,41^. Previous studies, however, suggest that perceptible drug effects (e.g., side-effects) can influence treatment effects in a particular direction e.g. leading to an overestimation of drug effects^40–42^. Relatedly, it remains to be determined whether the effects of repeated administration of psychedelic microdoses are different to those associated with a given active placebo^39^. Addressing this question is crucial for advancing on previous studies into microdosing, including RCTs that have employed inert placebos^36,37,70^.

In light of previous evidence^36,37,70,71,73^, the assumption that most people take microdoses that are too small to produce significant acute psychological or indeed pharmacological effects, does not seem reliable. Regardless of discernible psychoactivity, even a very small dose of a psychedelic could cause a functionally significant level of 5-HT2A receptor signalling, associated cortical plasticity, and increased context-sensitivity^19,74^—and one could argue that a low-level pharmacological effect and a positive expectancy/placebo effect are not mutually exclusive phenomena, and may, indeed, be synergistically interactive^42^. Relatedly, it remains entirely plausible, if not compelling, that non-pharmacological contextual factors influence microdosing outcomes^19,75,76^.

These are assumptions that the present study did not adequately address, but they are potentially fruitful avenues for future research^19^. The possibility of including appropriate active placebos that successfully maintain the integrity of study blinds is also worth considering. Combining subjective measures with objective physiological ones, using a multi-method approach^77^, would also hold merit^78^. Venturing into clinical populations rather than using samples of healthy volunteers, where there may be comparatively less scope for meaningful psychological change, is another worthy consideration.

Relatedly, almost ~ 50% of the present sample reported to have been diagnosed with one or more psychiatric disorders, suggesting its clinical relevance. The severity of self-reported depression and anxiety symptom scores approximated the normal/ healthy range within the first week of microdosing (Table 1), indicating a clinically meaningful, and quite rapid, improvement. Moreover, as one would expect, post-microdosing decreases in depression scores were greater in a subsample of participants who scored above a certain threshold for depression at baseline, and this effect occurred relatively rapidly, i.e. in the first week of microdosing (Supplementary Methods, Supplementary Figure S2). Thus, if the positive beneficial effects of microdosing are indeed ‘true effects’ (i.e. over-and-above mere placebo effects) microdosing may have a more rapid antidepressant effect than conventional antidepressant drugs – such as selective serotonin-reuptake inhibitors (SSRIs). The direct action of psychedelics at the 5-HT2A receptor may help explain such a scenario. In contrast, SSRIs have a delayed therapeutic action thought to be due to a gradual desensitisation of presynaptic autoreceptors—which ordinarily regulate post-synaptic serotonin release^79^.

In conclusion, the present study provides the first demonstration of the role of positive expectancy in mediating positive mental health outcomes associated with psychedelic microdosing, thus highlighting the need for caution in making claims about the therapeutic value of this practice. Awareness of design limitations help motivate and inform more rigorous studies to better test the effects of psychedelic microdosing in healthy and clinical populations.

## Supplementary Information

Supplementary Information.

## Data Availability

All data are available on reasonable request from the corresponding author.
