# Supplementary material for: Positive expectations predict improved mental-health outcomes linked to psychedelic microdosing
Source: Sci Rep. 2021 Jan 21;11:1941. doi: 10.1038/s41598-021-81446-7 (PMC7820236; doi:10.1038/s41598-021-81446-7)
Supplement: Supplementary file 1 — Supplementary Information. [file 41598_2021_81446_MOESM1_ESM.docx]

# Supplementary Information

# Title: Positive expectations predict improved mental-health outcomes linked to psychedelic microdosing

Kaertner, L. S.^1*^, Steinborn, M. B.^2^, Kettner, H. S.^1^, Spriggs, M. J.^1^, Roseman, L.^1^, Buchborn, T.^1^, Balaet, M.^3^, Timmermann, C.^1^, Erritzoe, D.^1^, Carhart-Harris, R. L.^1^,

^1^Centre for Psychedelic Research, Imperial College London

^2^Departmant of Psychology, Julius-Maximilans-University Wuerzburg

^3^Computational, cognitive and clinical neuroimaging laboratory, Imperial College London

**Correspondence:**

Laura Kaertner, M.Sc.

Centre for Psychedelic Research, Division of Brain Sciences

Imperial College London, W12 0NN London

e-mail: laura.kartner17@imperial.ac.uk

**Supplementary Table S1**

*Means, standard deviations, and Pearson correlation coefficients for baseline variables.*

| Variable | M | SD | 1 | 2 | 3 | 4 | 5 | 6 | 7 | 8 | 9 | 10 | 11 | 12 | 13 | 14 | 15 |
| --- | --- | --- | --- | --- | --- | --- | --- | --- | --- | --- | --- | --- | --- | --- | --- | --- | --- |
|  |  |  |  |  |  |  |  |  |  |  |  |  |  |  |  |  |  |
| 1. WEMWBS | 43.65 | 9.94 |  |  |  |  |  |  |  |  |  |  |  |  |  |  |  |
|  |  |  |  |  |  |  |  |  |  |  |  |  |  |  |  |  |  |
| 2. STAI | 48.70 | 14.61 | **-.74**** |  |  |  |  |  |  |  |  |  |  |  |  |  |  |
|  |  |  |  |  |  |  |  |  |  |  |  |  |  |  |  |  |  |
| 3. QIDS-SR_16_ | 9.25 | 5.73 | **-.78**** | **.74**** |  |  |  |  |  |  |  |  |  |  |  |  |  |
|  |  |  |  |  |  |  |  |  |  |  |  |  |  |  |  |  |  |
| 4. TIPI-E | 3.75 | 1.71 | **.39**** | **-.23**** | **-.26**** |  |  |  |  |  |  |  |  |  |  |  |  |
|  |  |  |  |  |  |  |  |  |  |  |  |  |  |  |  |  |  |
| 5. TIPI-A | 4.70 | 1.30 | **.30**** | **-.25**** | **-.24**** | **.14*** |  |  |  |  |  |  |  |  |  |  |  |
|  |  |  |  |  |  |  |  |  |  |  |  |  |  |  |  |  |  |
| 6. TIPI- C | 4.53 | 1.51 | **.34**** | **-.34**** | **-.37**** | .02 | **.19**** |  |  |  |  |  |  |  |  |  |  |
|  |  |  |  |  |  |  |  |  |  |  |  |  |  |  |  |  |  |
| 7. TIPI - ES | 4.08 | 1.61 | **.56**** | **-.71**** | **-.53**** | **.17**** | **.37**** | **.28**** |  |  |  |  |  |  |  |  |  |
|  |  |  |  |  |  |  |  |  |  |  |  |  |  |  |  |  |  |
| 8. TIPI- O | 5.79 | 1.07 | **.32**** | **-.16**** | **-.18**** | **.26**** | **.20**** | .07 | **.15*** |  |  |  |  |  |  |  |  |
|  |  |  |  |  |  |  |  |  |  |  |  |  |  |  |  |  |  |
| 9. BRS | 3.10 | 0.85 | **.55**** | **-.61**** | **-.49**** | **.34**** | **.31**** | **.20**** | **.57**** | **.18**** |  |  |  |  |  |  |  |
|  |  |  |  |  |  |  |  |  |  |  |  |  |  |  |  |  |  |
| 10. BEAQ | 46.30 | 12.69 | **-.58**** | **.48**** | **.56**** | **-.31**** | **-.34**** | **-.45**** | **-.42**** | **-.28**** | **-.48**** |  |  |  |  |  |  |
|  |  |  |  |  |  |  |  |  |  |  |  |  |  |  |  |  |  |
| 11. SCS | 29.56 | 11.72 | **.61**** | **-.50**** | **-.58**** | **.34**** | **.26**** | **.22**** | **.37**** | **.18**** | **.40**** | **-.44**** |  |  |  |  |  |
|  |  |  |  |  |  |  |  |  |  |  |  |  |  |  |  |  |  |
| 12. NR-6 | 3.92 | 0.88 | **.25**** | **-.14*** | **-.16**** | .01 | **.30**** | **.20**** | .10 | **.19**** | .08 | **-.29**** | .08 |  |  |  |  |
|  |  |  |  |  |  |  |  |  |  |  |  |  |  |  |  |  |  |
| 13. MODTAS | 38.25 | 16.63 | **.18**** | -.03 | -.01 | .09 | **.21**** | -.02 | -.03 | **.29**** | .06 | -.11 | .10 | **.43**** |  |  |  |
|  |  |  |  |  |  |  |  |  |  |  |  |  |  |  |  |  |  |
| 14. SSS | 42.65 | 9.58 | .04 | .09 | .07 | **.15*** | .07 | -.21** | -.01 | .03 | -.06 | **.20**** | .02 | -.12 | .11 |  |  |
|  |  |  |  |  |  |  |  |  |  |  |  |  |  |  |  |  |  |
| 15. PDI | 5.94 | 3.83 | -.03 | **.14*** | **.18**** | -.03 | -.01 | **-.22**** | **-.16**** | **.14*** | -.11 | **.23**** | -.10 | **.20**** | **.54**** | **.28**** |  |
|  |  |  |  |  |  |  |  |  |  |  |  |  |  |  |  |  |  |
| 16. Expectancy | 65.10 | 19.95 | **.13*** | -.11 | -.07 | .03 | **.15*** | .10 | .11 | **.16*** | **.13*** | -.04 | .03 | **.30**** | **.25**** | .02 | **.18**** |

*Note. M* and *SD* are used to represent mean and standard deviation, respectively. Pearson correlation coefficients are shown, * indicates *p* < .05. ** indicates *p* < .01.

Warwick-Edinburgh Mental Well-being Scale (WEMWBS); Quick Inventory of depressive Symptomatology (QIDS-SR_16_); six-item short-form of the state scale of the Spielberger State-Trait Anxiety Inventory (STAI-6); Ten-Item Personality Inventory (TIPI) with the subscales on (-E) Extraversion, (-A) Agreeableness, (-C) Conscientiousness, (-ES) Emotional Stability and (-O) Openness; Brief Resilience Scale (BRS); Brief Experiential Avoidance Questionnaire (BEAQ); Social Connectedness Scale (SCS); Nature Relatedness (NR-6); Modified Tellegen Absorption Scale (MODTAS), Peter’s Delusion Inventory (PDI) Short Suggestibility Scale (SSS); (Expectancy) Expectancy Score.

**Supplementary Table S2**

*Means, standard deviations and absolute frequencies of demographic data from survey one.*

| Total |  | 253 |
| --- | --- | --- |
| Gender | Male  Female  Other | 153 (60.5%)  99 (39.1%)  1 (0.4%) |
| Age |  | 35.47 (±11.87) |
| Nationality | United Kingdom  United States  Canada  Netherlands  Germany | 68 (26.9%)  56 (22.1%)  19 (7.5%)  27 (10.7%)  11 (4.3%) |
| Educational level | Left school before age 16 without qualifications  Some high school/GCSE level (in UK)  High school diploma/A-level education (in UK)  Some university (or equivalent)  Bachelor’s degree (or equivalent)  Post-graduate degree (e.g. masters or doctorate) | 2 (0.8%)  15 (5.9%)  36 (14.2%)  50 (19.8%)  90 (35.6%)  60 (23.7%) |
| Employment status | Student  Part-time job  Full-time job  Retired  Unemployed  Disabled, not able to work | 41 (16.2%)  36 (14.2%)  145 (57.3%)  2 (0.8%)  15 (5.9%)  14 (5.5%) |
| Psychiatric history | Ever been diagnosed with at least one psychiatric illness  Never been diagnosed with a psychiatric illness | 117 (53.8%)  136 (46.2%) |
| Previous psychedelic drug use | Never (psychedelic naive)  once  2-5 times  6-20 times  More than 21 times | 38 (15.0%)  34 (13.4%)  54 (21.3%)  72 (28.4%)  55 (21.7%) |
| Microdosing experience | yes  no | 75 (29.6%)  178 (70.4%) |

*Note:* Means ± standard deviations and absolute frequencies are illustrated. Numbers in parenthesis indicate the percentages corresponding to the absolute frequencies.

^a^ Only the five most common nationalities are reported. ^b^ Diagnosed with at least one of the specified psychiatric illnesses. Multiple responses were possible to account for comorbidity and/or multiple diagnoses throughout life.

#

**Supplementary Figure S1.** Four items assessing participants‘ attitude towards psychedelic drugs measured in the first survey.

**Supplementary Table S3**

*Microdosing parameters.*

| Timepoint |  | Baseline | Week 1 | Week 2 | Week 3 | Week 4 |
| --- | --- | --- | --- | --- | --- | --- |
| Drug Type | Psilocybin/Magic Mushrooms | 121 (47.82%) |  |  |  |  |
|  | LSD/ 1P-LSD | 106 (41.9%) |  |  |  |  |
|  | DMT/ 5-MeO-DMT | 2 (0.8%) |  |  |  |  |
|  | Mescaline | 3 (1.2%) |  |  |  |  |
|  | Ibogaine | 4 (1.6%) |  |  |  |  |
|  | Mixed substances | 17 (6.7%) |  |  |  |  |
|  | Other |  |  |  |  |  |
| Drug dose ^a^ | Tiny microdose |  | 26 (10.3%) | 13 (5.1%) | 16 (6.3%) | 7 (2.8%) |
|  | Small microdose |  | 78 (30.8%) | 59 (23.3%) | 49 (19.4%) | 43 (17.0%) |
|  | Moderate microdose |  | 41 (16.2%) | 29 (11.5%) | 22 (8.7%) | 19 (7.5%) |
|  | Moderate/ ‘high microdose‘ |  | 12 (4.7%) | 9 (3.6%) | 10 (4.0%) | 8 (3.2%) |
|  | ‘High‘ microdose |  | 1 (0.4%) | 1 (0.4%) | 1 (0.4%) | 1 (0.4%) |
|  | ‘Very high‘ microdose |  | 4 (1.6%) | 4 (1.6%) | 4 (1.6%) | 3 (1.2%) |
| Drug effects | Definitely no detectable effects |  | 14 (5.5 %) | 17 (6.7%) | 14 (5.5%) | 8 (3.2%) |
|  | I could just be imagining them ^b^ |  | 39 (15.4%) | 29 (11.5%) | 34 (13.4%) | 27 (10.7%) |
|  | Possible mild effects |  | 45 (17.8%) | 28 (11.1%). | 25 (9.9%) | 22 (8.7%) |
|  | Mild but quite noticeable effects |  | 39 (15.4%) | 31 (12.3%) | 26 (10.3%) | 18 (7.1%) |
|  | Clearly noticeable effects |  | 25 (9.9%) | 9 (3.6%) | 3 (1.2%) | 6 (2.4%) |
|  | Stronger than typical 'microdose' level effects |  | 0 | 1 (0.4%) | 0 | 0 |
| Dosing days | 1 |  | 11 (4.3%) | 10 (4.0%) | 13 (5.1%) | 16 (6.3%) |
|  | 2 |  | 59 (23.3%) | 69 (27.3%) | 61 (24.1%) | 37 (14.6%) |
|  | 3 |  | 77 (30.4%) | 28 (11.1%) | 19 (7.5%) | 23 (9.1%) |
|  | 4 |  | 10 (4.0%) | 7 (2.8%) | 7 (2.8%) | 3 (1.2%) |
|  | 5 |  | 4 (1.6%) | 1 (0.4%) | 1 (0.4%) | 0 |
|  | 6 |  | 0 | 0 | 0 | 1 (0.4%) |
|  | 7 |  | 1 (0.4%) | 0 | 1 (0.4%) | 1 (0.4%) |
| Total |  | 253 | 162 | 115 | 102 | 81 |

*Note.* Absolute frequencies and the percentages are shown. Drug type was assessed at baseline.

^a^ Drug doses were reported in LSD equivalents: 1-5 mcg or ~1/20 of a tab max, LSD reference: 6-10 mcg or ~1/10 of a tab max, LSD reference: 11-15 mcg or ~1/7 of a tab max, LSD reference: 16-20 mcg or ~1/4 of a tab max, LSD reference: 21-30 mcg or ~1/3 of a tab max, LSD reference: 31+ mcg or more than 1/3 of a tab max.

^b^ *“Effects so slight, I could just be imagining them”.*

**Supplementary Table S4**

*Descriptive statistics for secondary outcome variables measured at baseline and key-endpoint.*

|  | Baseline |  | Endpoint |  |
| --- | --- | --- | --- | --- |
| Variables | *M* | *SD* | *M* | *SD* |
| BRS | 3.22 | .85 | 3.51 | .74 |
| BEAQ | 43.67 | 12.85 | 39.83 | 12.45 |
| SCS | 30.15 | 12.42 | 35.54 | 11.92 |
| NR-6 | 3.99 | .84 | 3.99 | .84 |
| TIPI-E  TIPI-A  TIPI-C  TIPI-ES  TIPI-O | 3.75  4.63  5.03  4.23  5.77 | 1.73  1.29  1.38  1.67  1.15 | 3.77  5.00  5.23  4.69  5.85 | .1.5  1.12  .1.21  1.42  .96 |
| MODTAS | 38.52 | 15.74 | 40.22 | 15.44 |
| PDI | 5.54 | 3.76 | 5.10 | 3.55 |
| SSS | 40.38 | 8.97 | 39.54 | 9.37 |

*N*=81. Descriptive statistics are shown, *M* = means and *SD* = standard deviation. Brief Resilience Scale (BRS); Brief Experiential Avoidance Questionnaire (BEAQ); Social Connectedness Scale (SCS); Nature Relatedness (NR-6); Ten-Item Personality Inventory (TIPI) with the subscales on I Extraversion, (A) Agreeableness, (C) Conscientiousness, (ES) Emotional Stability and (O) Openness; Modified Tellegen Absorption Scale (TAS), Peter’s Delusion Inventory (PDI) Short Suggestibility Scale (SSS).

**Supplementary Table S5**

|  | Week 1 | Week 2 | Week 3 | Week 4 |
| --- | --- | --- | --- | --- |
| Change in relation to baseline  **Positive items** |  |  |  |  |
| - My ability to cry | .10 ± .81 | .40 ± .88 | .35 ± .71 | .46 ± .82 |
| - My ability to feel compassion | .81 ± .78 | .91 ± .89 | .91 ± .84 | .93 ± .87 |
| - My ability to feel intense emotion | .43 ± .81 | .60 ± .83 | .62 ± .71 | .76 ± .79 |
| - Creative feelings | .96 ± .94 | .87 ± .90 | .88 ± .86 | .93 ± .97 |
| - Feelings of pleasure | .85 ± .80 | .87 ± .93 | .91 ± .93 | .90 ± .83 |
| - My ability to concentrate | -.12 ± .91 | .07 ± .68 | .01 ± .61 | .06 ± .90 |
| **Negative items** |  |  |  |  |
| - A problematic restlessness | .04 ± .76 | .12 ± .47 | .06 ± .52 | .01 ± .53 |
| - Feeling manic | .59 ± .95 | .47 ± .98 | .40 ± .96 | .44 ± .98 |
| - Feeling paranoid | -.18 ± .52 | -.07 ± .50 | -.09 ± .51 | -.13 ± .42 |
| - Headaches | .06 ± .54 | .03 ± .65 | .04 ± .61 | .09 ± .48 |
| - Troubling visual perceptual changes | .10 ± .31 | .13 ± .45 | .04 ± .21 | .10 ± .35 |
| - Gut problems, e.g. diarrhea | .15 ± .47 | .06 ± .45 | .06 ± .38 | .16 ± .56 |
| - Dizziness | .10 ± .31 | .07 ± .26 | .06 ± .29 | .06 ± .24 |
| - Nausea/vomiting | .04 ± .21 | .06 ± .29 | .06 ± .24 | .04 ± .21 |
| - Sweating | .12 ± .37 | .13 ± .644 | .04 ± .47 | .06 ± .42 |
| - Dry mouth | .15 ± .36 | .09 ± .29 | .06 ± .24 | .09 ± .29 |
| - Drowsiness | .01 ± .80 | -.07 ± .56 | .04 ± .47 | -.06 ± .52 |

*Weekly scores on the Post Treatment Change Scale (PTCS)*

*Note.* Means ± standard deviations are shown. The PTCS was assessed weekly by the end of each week (except for baseline).

Below is the complete PTCS.

Please rate each of the following 17 items according to how you have felt on microdosing days over the last week. Rate on average if you have microdosed on more than one day.

Please give your rating based on whether you have noticed any change in relation to your usual/baseline state, i.e. before beginning your microdosing routine.

1) My ability to cry

2) My ability to feel compassion

3) My ability to feel intense emotion

4) Creative feelings

5) Feelings of pleasure

6) A problematic restlessness

7) Feeling manic*

8) My ability to concentrate

9) Feeling paranoid

10) Headaches

11) Troubling visual perceptual changes**

12) Gut problems, e.g. diarrhoea

13) Dizziness

14) Nausea/vomiting

15) Sweating

16) Dry mouth

17) Drowsiness

Discrete scale = 7 increments

-3 = Greatly reduced

-2 = Moderately reduced

-1 = Slightly reduced

0 = No change or not present

+1 = Slightly increased

+2 = Moderately increased

+3 = Greatly increased

* [WEB LINK TO THIS] The standard psychiatric definition of mania is “a distinct period of abnormally and persistently elevated, expansive, or irritable mood.” It may feature any of the following:

1) Inflated self-esteem or grandiosity.

2) Decreased need for sleep (e.g. feeling rested after only three hours of sleep).

3) More talkative than usual or pressure to keep talking.

4) Flight of ideas or subjective experience that thoughts are racing.

5) Distractibility (i.e. attention too easily drawn to unimportant or irrelevant external stimuli), as reported or observed.

6) Increase in goal-directed activity (either socially, at work or school, or sexually) or psychomotor agitation (i.e. purposeless non-goal-directed activity).

7) Excessive involvement in activities that have a high potential for painful consequences (e.g. engaging in unrestrained buying sprees, sexual indiscretions, or foolish business investments).

** [WEB LINK TO THIS] By “troubling perceptual changes” we are referring to any of the following that cause you significant distress: halos or auras surrounding objects, trails following objects in motion, difficulty distinguishing between colors, apparent shifts in the hue of a given item, the illusion of movement in a static setting, air assuming a grainy or textured quality (visual snow or static), distortions in the dimensions of a perceived object, and/or a heightened awareness of floaters.

# Supplementary Methods: depressive symptomatology

In order to test whether the observed changes in QIDS-SR_16_ scores differed between the two subsamples (depressed vs. non-depressed), the sample was split based on QIDS-SR_16_ interpretation norms (< 5 = no depression), resulting in *n* = 28 (non-depressed) and *n* = 40 (depressed) participants per group. A mixed between-within ANOVA was conducted. Square root transformation of QIDS-S16 scores was applied due to violations of homogeneity of variances. Greenhouse-Geisser correction was employed as adjustive method. Overall, results of the mixed ANOVA revealed a significant main effect of time [*F*(3.2, 210.4) = 34.247, *p* < .001, *η_p_2* = .342] and group [*F*(1, 66) = 52.128, *p* < .001, *η_p_2* = .441]. The interaction term between time and group [*F*(3.2, 210.4) = 11.077, *p* < .001, *η_p_2* = .144] was significant, indicating that the change over time differed between the groups (Supplementary Figure 2).

In order to further explore the effect of time separately per subsample, simple effects analyses on the untransformed data (separate repeated measures ANOVAs for each group) were conducted. Results revealed a significant main effect of time on QIDS-SR_16_ scores in the group classified as depressed (*n* = 40) [*F*(2.4, 92) = 56.146, *p* < .001, *η_p_2* = .59]. This decrease was most pronounced at the second and third time-point (week 1 and 2) but quickly reached asymptote afterwards. The planned contrast analysis (simple) showed significant differences between well-being scores at baseline and all successive timepoints: week 1 [*F*(1, 39) = 68.271, *p* < .001, *η_p_2* = . .64], week 2 [*F*(1, 39) = 71.634, *p* < .001; *η_p_2* = .65], week 3 [*F*(1, 39) = 84.883, *p* <. 001; *η_p_2* = .69] and week 4 [*F*(1, 39) = 100.162; *p* < .001, *η_p_2*= .72]. For the non-depressed group (*n* = 28), the main effect of time on QIDS-SR_16_ scores was not significant [*F*(2.6, 71.5) = 2.242, *p* < .098, *η_p_2* = .08]. Inspection of the planned contrast analysis revealed a significant decrease for week 1 [*F*(1, 27) = 5.094, *p* < .032, *η_p_2* = .16], week 2 [*F*(1, 27) = 5.132, *p* < .032, *η_p_2* = .16] and week 3 [*F*(1, 27) = 10.016, *p* < .098, *η_p_2* = .27], which was not sustained at week 4 [*F*(1, 27) = 3.003, *p* < .098, *η_p_2* = .10]. These results demonstrate that the effects of microdosing differed depending on the classification as depressed vs. non-depressed, showing that the antidepressant effect is more pronounced in the depressed group. This may be due to the fact that the depressed group has more scope for change to begin with, or that individuals with symptoms of mood disorders may experience different antidepressant effects than individuals who score in the healthy range.

**
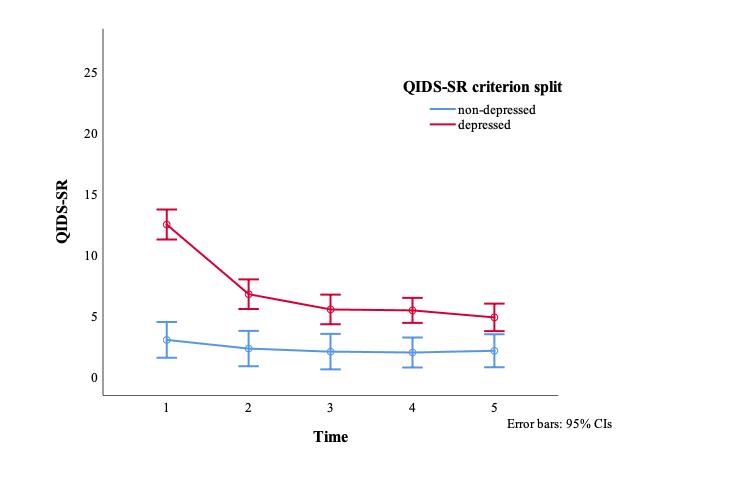
 Supplementary Figure S2**

Mean QIDS-SR scores over all time-points are shown for individuals in the depressed range (QIDS–SR > 5) and non-depressed range (QIDS-SR < 5). Error bars represent 95% confidence intervals.

**Supplementary Methods:** attrition analysis

In order to determine whether there was a sample bias due to the drop out, Mann-Whitney U and Chi-square tests were conducted to determine whether there were baseline differences in demographic and trait-variables between participants who dropped out before completing the study (n = 172) and participants who completed the study endpoint (n = 81). The variables were chosen based on theoretical considerations. Mann-Whitney U tests were conducted to test for differences in age, expectancy scores and trait conscientiousness. Results revealed that participants who dropped out had significantly lower scores in the personality trait conscientiousness [*Md* = 4.50, *U* = 4993.0 *p* < .001] and were of younger age [*Md* = 32, U = 5543.5 *p* = .009] as compared to participants who completed the study (*Md* = 5.00, *Md* = 37.00). No significant differences were found in baseline expectancy scores between participants who dropped out (*Md* = 63.62) and participants who completed the last time-point (*Md* = 69.86), *U* =6003.0, *p* = .076.

Chi-Square tests of independence (N = 253) revealed no association between drop-out (*0 = completed, 1 = drop out)* and the following variables: gender (*0 = male, 1 = female:* χ2(1) = .11, *p* = .745), employment (χ2(1) = 7.15, p = .13), lifetime psychiatric history (*0 = none, 1 = reported to have been diagnosed:* χ2(1) = 3.05, *p* = .81), having prior experience with psychedelics at the begin of the study (*yes/no:*χ2(1) = .04, *p* = .848), being an active advocate of psychedelic drug use (χ2(1) = .00, *p* = .99) or being an active advocate of the therapeutic use of psychedelics (χ2(1) = .22, *p* = .640). For the latter two items the negatively and neutral (*“Strongly Disagree”, “Disagree”, “Neither Agree Nor Disagree”*) and the positively formulated items (*“Agree”* and *“Agree Strongly”*) were merged into one category, respectively, resulting in one positive versus one neutral/negative category. Changes of STAI-6 scores in week 1 (week 1 – baseline) were coded as 0 = increase and 1 = decrease or no change were not significantly associated with drop out at week 4 (χ2(1) = .56, *p* = .454).

**Supplementary Methods:** participant information sheet and consent form

**Thank you for your interest in the Microdosing Survey Study - a web-based study to assess the subjective effects of microdosing psychedelic substances**

***Are you planning to microdose a psychedelic substance (e.g. LSD/ 1P-LSD, psilocybin, ayahuasca/DMT, etc.) in the near future? Your experience is very valuable to us, so please sign up & take part in our study!***

The *Microdosing Survey Study* is being run by the Psychedelic Research Group at Imperial College London, led by Professor David Nutt and Dr Robin Carhart-Harris. The aim of this project is to collect data on the process of microdosing a classic psychedelic substance (i.e. psilocybin, LSD/1P-LSD, ayahuasca, AL-LAD, salvia divinorum, DMT, 5-MeO-DMT, iboga/ibogaine or mescaline). Specifically, we aim to use online questionnaires to collect relevant data at different time points, about one week before, once a week during your microdosing experience (preferably 4-6 weeks), 6 weeks after and 6 and 12 months after the begin your experience. If you intend to microdose longer than 4-6 weeks, you are still eligible to take part.

The data we collect from this study will help to advance the scientific understanding of microdosing psychedelics, including how this experience can lead to the best possible outcomes. Further we intend to collect important information about best practice, safer use and harm reduction. The project has the potential to help develop future controlled studies on psychedelics in terms of microdosing, including clinical trials. We greatly appreciate the time you dedicate to this project. The data you provide is very valuable to us. We protect your anonymity, as no personally identifying information will be collected and we will not collect your IP address. As the *Microdosing Survey Study*is web-based, internet access is required. We further want to inform you that you will receive the results of the study as soon as the data is analysed.

**Participation**

To take part, you must be at least 18 years old of age, have a good understanding of the English language, and plan to microdose a **classic psychedelic drug (i.e. psilocybin, LSD/1P-LSD, ayahuasca, AL-LAD, salvia divinorum, DMT, 5-MeO-DMT, iboga/ibogaine or mescaline)**anytime within the next months. You are eligible to participate whether you had any experience with psychedelic substances or not and as well if you are already actively microdosing, there are no further exclusion criteria. You must also be willing to provide us with a correct E-mail address and be willing to receive a small number of E-mail reminders, which remind you to complete the relevant questionnaires at the appropriate time-points. If you decide to take part you should indicate your agreement to the online consent form and provide your e-mail address and your intended start date.

**Procedure**

We will ask you to complete online questionnaires on nine different time points. We will assess your general state (baseline) **one week before you** start your microdosing experience. Further you will receive questionnaires **once a week during**a 4-6 week microdosing experience. In week 5 you will receive another weekly questionnaire together with the possibility to complete an open report, where you can describe your experience in your own words. After this period you will receive follow-up questionnaires at 6 weeks after, 6 & 12 months after the begin of your experience. *If you are planning to microdose for onger than 4 weeks, you are still eligible to take part, as we will assess your ongoing experience in the weekly questionnaires.*

**Time-point 1: Anytime within 1 week prior to the planned start of your microdosing experience**

If you plan to start microdosing a psychedelic within one-week from now, then this time-point may be today. It is just important that you start microdosing within 1 week of completing the baseline questionnaire. It is also very important for us that you complete the questionnaires at the 7 subsequent time-points (see below).

If after completing this questionnaire, you later decide to change your plans and either delay or cancel your planned psychedelic experience, this is fine. Completing this questionnaire does not oblige you to perform the microdosing routine. It will take approximately 40 minutes to complete the questionnaire at time-point 1. If you are not able to complete the questionnaire in one run, you can save your progress and complete it at another moment.

3 days before your start you will receive a protocol sheet for you to print out, where you can note your dosing day/s and the amount of substance you used on that day/s.

**Time-point 2, 3, 4, 5: measures once a week (first weekly questionnaire starts 1 week after you started dosing)**

The questionnaire at time-points 2, 3, 4, 5 are weekly questionnaires and should be completed once a week during the 4-week period. This means that you will receive the first weekly questionnaire 7 days after your first microdose. The weekly questionnaires should be completed on the day you receive them. It will take approximately 20 minutes to complete. We will send you an E-mail reminder about the upcoming questionnaires based on the start date you provide to us for your planned microdosing experience. Try to be accurate with this date and please inform the research team if the date changes (email: info@microdosingsurvey.com).

**Time-Point 6: 5 weeks after & open Report**

In week 5 you will receive a weekly questionnaire together with the opportunity to complete an open report about your experience. Here you can share your experience with us in further detail.

**Time-point 7: 6 weeks after the begin of your microdosing experience**

The questionnaire after the microdosing experience (time point 6) should be completed 6 weeks after the start your experience. If this is not possible, please try to complete it as soon as possible. It will take about 35 minutes to complete. If you are not able to complete the questionnaire in one run, you can save your progress and complete it at another moment. Please then try not to let 7 days pass before completing this questionnaire.

**Please do go on completing these questionnaires, as if you drop out early, this affects our ability to use your data!**

**Time-point 8 and 9: 6 and 12 months after your microdosing experience**

We would ideally like to follow you up after time-point 6 (6 weeks after) to assess how you are doing for a longer period, but we acknowledge that you might wish to withdraw from receiving email reminders about this follow-up. You are free to withdraw from these email reminders at any point. Please keep in mind, that **any data you provide is valuable for us** and we **would appreciate if you continue the follow-up measures**as well.

At the end of every questionnaire there will be a response confirmation: “You are about to submit your response. Are you still happy for us to use your anonymous data for our research?” An answer of “yes” will automatically submit the form.

**Important information**

**Safety**

At this point we want to emphasize that we are not encouraging drug taking behaviour, but if you were generally planning to microdose in the near future, we welcome you as a participant and appreciate your participation in our study.

You can freely choose how to arrange your microdosing experience, but we recommend that you do sufficient research before you start microdosing in order to ensure a safe use of those substances. We won't provide information about where you can find the required substances or about how to administer them.

**Anonymity**

All obtained data is anonymous, no data will be shared that will identify you or your responses. You can withdraw from this project at any time by clicking “unsubscribe me from this mailing list” in the E-mails you receive from us, the participation in this study is non-binding and voluntary. If you wish that your previously submitted data will be discarded, please contact the research team via info@microdosingsurvey.com and request the deletion of your data.

**Sign-up information**

If you are about to sign up, please make sure that you provide your **correct E-mail address**, as you won't receive the questionnaires otherwise.

If your intended start date is in less than 7 days from today, please make sure to sign up for the first possible start date you can pick. After this, contact the research team via info.@microdosingsurvey.com and inform the study administrator of your intended start date, so we can adapt it manually and you receive the necessary questionnaires at the right time-point.

## **Sign Up**

Your first name

Email address

Date of first microdose

**Study Terms**

I have read and understand the information provided.

I agree to take part in the study.

I understand that my participation is voluntary and that i am free to withdraw at any time without giving any reason and without my legal rights being affected.

I agree to my anonymised data being stored on password protected Imperial College London computer systems.

I agree to my email being retained.
